# HPV infection and P16 expression in oral and oropharyngeal cancer in Kazakhstan

**DOI:** 10.1186/s13027-018-0175-8

**Published:** 2018-01-12

**Authors:** Dauren Adilbay, Galim Adilbayev, Gulzhan Kidirbayeva, Viktoria Shipilova, Zhanat Sadyk, Gulsum Koyanbekova, Ekaterina Sokolenko, Jan Klozar

**Affiliations:** 1 Kazakh Institute of Oncology and Radiology, 91 Prospekt Abaya, Almaty, Kazakhstan 050022; 2 Head and neck oncology center, Kazakh Institute of Oncology and Radiology, Almaty, Kazakhstan; 3 Pathology and molecular biology department, Kazakh Institute of Oncology and Radiology, Almaty, Kazakhstan; 4 Charles University in Prague, University Hospital Motol, Prague, Czech Republic

**Keywords:** HPV, Oral cavity, Oropharyngeal cancer, p16 IHC, Kazakhstan, This research was presented as an E-poster on IFOS 2017 ENT world congress (24–28 June) Paris, France. Abstract № HN-HPV-20.

## Abstract

**Background:**

Human papillomavirus (HPV) is an important etiologic factor in different cancers of anogenital region and also in a fraction of head and neck cancers (HNC) particularly oropharyngeal tumors. The HPV16 genotype associated with the majority of HPV-related head and neck carcinomas. Th incidence of oropharyngeal cancer is increasing in many countries, and the rate of HPV positive tumors is about 70% in Europe and North America. Little known about the prevalence of HPV in HNC in Central Asia.

**Methods:**

It’s a prospective analysis of patients with verified oral or oropharyngeal cancer. Sociodemographic and clinical data obtained on admission to treatment. The diagnosis of HPV positivity assessed by both the P16 expression on immunohistochemistry(IHC) and polymerase chain reaction (PCR)with HPV DNA detection and HR HPV type determination.

**Results:**

Seventy six patients with oral and oropharyngeal cancer tested for HPV. Forteen cases were positive for HPV by PCR and 15 cases by P16 IHC. Of the 35 oropharyngeal tumors, nine were HPV DNA and p16 IHC positive, giving the rate of 25.7%. Of the 41 oral tumors, five were HPV DNA and six p16 IHC positive, giving the rate of 12.2%.

**Conclusion:**

It is the first study mapping prevalence of HPV positivity in oral and oropharyngeal cancer in the Central Asian region. The rate of HPV positivity was higher in oropharyngeal than in oral cancer, the nonsmokers were significantly more frequent in the HPV positive group and HPV 16 was the most frequent type. However, the HPV positivity rates are lower than referred in the western world.

## Background

Oral cavity and oropharynx cancer is a major global health issue, they both constitute the majority of head and neck cancers. Globally 300,000 men and 130,000 women get diagnosed with these diseases annually, and the annual mortality is 160,000 and 68,000 respectively [1].

Main predisposing factors are smoking and alcohol consumption [2]. Despite the decrease in the incidence of tobacco-induced cancers in many countries, there is a rising incidence of oropharyngeal cancer worldwide. It is due to the increasing proportion of HPV-induced particularly oropharyngeal head and neck cancers [3, 4].

It’s known that HPV is an important etiologic factor in oropharyngeal cancer, yet, its role in other head and neck sites is unclear. There are more than a dozen of high-risk HPV types that can cause cancer. The 13 most frequent types of high-risk HPV (HR HPV) that are 16, 18, 31, 33, 35, 39, 45, 51, 52, 56, 58, 59 and 68 [5, 6]. Types 16 and 18 are the most frequently detected in cervical as well as in oropharyngeal cancers [5]. Most HR HPV positivity rate referred in up to 23% in oral cavity cancer and 24% in laryngeal cancer, but which is much less than in oropharyngeal cancer, where 50–60% of cases are HPV positive [6–8]. HPV-associated oropharyngeal cancer has unique morphologic features [9, 10]. These tumors are non-keratinizing squamous cell carcinomas with basaloid appearance and hyper-expression of p16. In contrast, the HPV negative tumors are usually keratinizing and consist of polygonal cells [9]. Worldwide incidence of these cancers ranges from 14% to 85% depending on the region [1, 11, 12]. From a clinical point of view, the most important difference is the better prognosis of patients with HPV positive cancers. This data led to the change of TNM classification for HPV positive tumors and considerations about treatment de-escalation [13, 14].

Kazakhstan is a multi-ethnic country located in Central Asia with a population of 17 million people. Its population is well known for high level of diversity, due to forced translocation of many nations during the Soviet period and a mixed structure of Kazakh nation [15]. The variety of population makes the possibility to get exciting results regarding the overall prevalence of HPV in the community, the distribution of HPV types and possibly also the relation to ethnicity. Up to date, there is only one published paper mapping the HPV prevalence in PAP smears of Kazakhstan women [16]. Each year more than 700 patients are diagnosed with oral cavity and oropharyngeal cancer in Kazakhstan [17, 18], and up to date, no studies were investigating HPV-associated head and neck cancer.

The role of HPV in cancerogenesis is now well documented, and the biological and clinical differences between HPV positive and negative tumors will probably very soon have consequences also in treatment approach [19]. From this point, it is essential to get the data of prevalence and differences in ethnic-geographic distribution in Kazakhstan. It will give the possibility to plan awareness campaigns and provide personalized treatment to patients in this country.

## Methods

It is a prospective analysis of patients with verified oral or oropharyngeal cancer referred to a single tertiary institution (observational study). Sociodemographic and clinical data obtained through a questionnaire on admission to treatment. Approval for the study received from the local institutional ethics committee, and informed consent obtained from all study participants.

The study enrolled 76 patients with oral and oropharyngeal cancer treated between February 2015 and May 2017 in Kazakh Institute of Oncology and Radiology. All patients have squamous cell carcinoma of different grades. Out of 76 patients, 35 had oropharyngeal cancer and 41 oral cavity cancer.

Part of the fresh tumor tissue from a biopsy specimen taken for PCR analysis and p16 IHC staining performed on formalin fixed paraffin embedded slices.

### HPV-DNA PCR

RIBO-sorb nucleic acid extraction kit was used to extract and purify DNA from cancer tissue. RIBO-sorb nucleic acid extraction kit is reagents kit for rapid and efficient manual extraction and purification of DNA from various biological materials.

AmpliSens HPV HCR genotype-FRT PCR kit is an in vitro nucleic acid amplification test for qualitative detection and differentiation of high risk (HR) human papillomaviruses (HPV) types 16, 18, 31, 33, 35, 39, 45, 51, 52, 56, 58, 59 DNA in the clinical material by using real-time hybridization-fluorescence detection of amplified products [5].

The test based on simultaneous PCR (multiplex-PCR) and real-time detection of three HPV types and b-globin gene DNA, used as internal control, in one tube. The analysis of 12 HPV types is carried out in four tubes. Each HPV type registered on its channel that allows not only to detect but also to differentiate the virus genotype. The DNA target selected as an endogenous internal control is a human genome fragment. AmpliSens HPV HCR genotype-FRT PCR kit uses “hot-start,” which significantly reduces the frequency of nonspecifically primed reactions.

### p16 immunohistochemistry

p16 IHC was carried out using the CINtec Histology kit (mtm Laboratories, AG, Germany) on a Ventana Benchmark Autostainer. p16 IHC was scored as positive if there was strong and diffuse nuclear and cytoplasmic staining present in greater than 70% of the malignant cells. All other staining patterns scored as negative. All samples were scored independently by two expert pathologists, and discordant cases were reviewed to come to a consensus score. Examinations were carried out at the Department of Pathology of Kazakh Institute of Oncology and Radiology.

## Results

There were 76 patients with oral and oropharyngeal cancer enrolled in the study. Of them 35 had oropharyngeal and 41 oral cavity cancer, 50 were male and 26 female. Mean age was 57.2 years (standard deviation - 11.45 years), with youngest and oldest participant were 26 and 79 respectively. Patients characteristics presented in Table 1.

**Table 1:** Patients characteristics

Primary Site	II stage	III stage	IV stage	Male	Female	Total
Oropharyngeal cancer	10 (28.6%)	19 (54.3%)	6 (17.1%)	25 (71.4%)	10 (28.6%)	35 (46.1%)
Tonsillar	5	10	3	13	5	18 (51.4%)
Base of tongue	3	3	0	4	2	6 (17.1%)
Other	2	6	3	8	3	11 (31.4%)
Oral cavity cancer	12 (29.3%)	25 (61.0%)	4 (9.8%)	25 (61%)	16 (39%)	41 (53.9%)
Floor of mouth	5	6	2	10	3	13 (31.7%)
Tongue	5	16	2	14	9	23 (56.1%)
Buccal mucosa	1	1	0	0	2	2 (4.9%)
Alveolar ridge, retromolar trigone	1	2	0	1	2	3 (7.3%)

Of the 76 patients, 14 cases were positive for HPV by PCR and 15 by P16 IHC. One patient had positive p16 IHC and negative PCR test. Of the 35 oropharyngeal tumors, nine were HPV DNA and p16 IHC positive, giving the rate of 25.7%. Out of nine positive cases, four are tonsillar cancers and two base of tongue cancers, what corresponds with the distribution of the whole group of oropharyngeal tumors.

Of the 41 oral tumors, five were HPV DNA positive and six patients were p16 IHC positive, giving the rate of 12.2% (excluding the P16 only positive case). Three of them are tongue cancer, one floor of the mouth and one alveolar ridge tumors. All three tongue cancers were located closer to posterior third of the tongue and even with extension to the base of the tongue.

HPV 16 was the most prevalent type in both the oropharyngeal and oral cavity cases, other types found were 18 and 56, one case each (Table 2).

**Table 2:** HPV-induced cancer prevalence and types detected

Primary Site	P16+ (IHC)	HPV +			HPV -	HPV+ prevalence	95% CI (Wilson)		Total
		HPV16	HPV18	HPV56			Lower	Upper	
Oropharyngeal cancer	9	8	1	0	26	0.257	0.141	0.421	35
Oral cavity cancer	6	4	0	1	36	0.122	0.053	0.255	41
All sites	15	12	1	1	62	0.184	0.113	0.286	76

Smoking was less prevalent in the group of HPV positive patients, two had a history of smoking out of 14, comparing 40 out of 62 in the HPV negative group of patients. The difference is statistically significant with *p* = 0.0018 (Chi-squared test).

In the oropharyngeal cancer group, 25 patients had advanced tumors stage III and IV. Patients with oropharyngeal cancer received concurrent chemoradiotherapy (CRT) according to the local standards. The local CRT standard includes delivering IMRT radiotherapy to tumor and neck with a concurrent infusion of platinum-based agent weekly or every three weeks.

In the group of oral cancers, 29 out of 41 had advanced oral cancer stage III and IV. They were treated mostly by surgery and adjuvant RT or CRT for non-operable cases. Adjuvant CRT in the oral cavity cancer group is used only for cases with adverse features on permanent pathology.

## Discussion

Cancers of the head and neck is a heterogeneous group of tumors with various anatomic sites with different etiologic factors. Tobacco exposure (either via active or passive smoking as well as consumption of smokeless tobacco) is the leading risk factor for head and neck cancers [2]. But in many developed nations with decreasing tobacco usage and consequently decreasing the incidence of tobacco induced cancers the incidence of oropharyngeal cancer is rising. This development attributed to the spread of HR HPV infection [1, 20, 21]. The data gathered in different multinational trials confirms the need for evaluation of HPV prevalence by region and country, as there are important differences between patient groups from Western Europe, Eastern Europe, and Asia [3]. As mentioned in many systematic reviews and meta-analysis one of the main limitations for comparing different studies was the lack of uniformity of the tests used [8]. In this study, we attempt to overcome this limitation by the use of commercial kits for both the p16 ICH and HPV DNA detection and type determination.

Kazakhstan is a partly Eastern European, partially Asian state with a mixed population. The primary risk factor for the local population is the smoking, as there is no or very low level of smokeless tobacco consumption prevalent in other Asian countries. The incidence of HNC is low in Kazakhstan when compared with, e.g., Europe. However, we can follow similar trends. There is rising age-standardized incidence rate of oral and oropharyngeal cancer from 3.8 in 2004 to 4.7 in 2015 per 100,000. In the same time incidence of laryngeal cancer mostly attributed to smoking has decreased from 2.6 in 2004 to 2.3 in 2015 [22]. Similarly, as in other countries this trend can be attributed to rising number of HPV induced oropharyngeal cancer, but further studies needed to establish the liaison, as some trials show the stable percentage of HPV positive oropharyngeal cancers over a decade [23]. In our study, most of the positive cases are from the oropharynx. Even several cases from oral cavity are from sites anatomically closer to the oropharynx, like the posterior third of the tongue or mandibular alveolar ridge. This site dependence is the same as described in many trials performed worldwide [1, 3].

## Conclusion

In summary in this first study in the Central Asian region the prevalence of HPV induced oral and oropharyngeal cancer is lower than in Europe and North America, but higher than in other Asian countries [3, 21]. In concordance with European and American results, the rate of HPV positivity was higher in oropharyngeal than in oral cancer, the nonsmokers were significantly more frequent in the HPV positive group and HPV 16 was by far the most frequent type. Further studies are needed to establish a better understanding of HPV prevalence and time trends in head and neck cancers in Kazakhstan. This trial provides the first valuable information for public policy and healthcare workers to plan screening, awareness campaigns, vaccination and in the future also the treatment of these tumors.
